# Long-Term Follow-Up of a Female Patient Treated with Olaparib—Hope for a Long Life without Relapse?

**DOI:** 10.3390/ijerph18073430

**Published:** 2021-03-26

**Authors:** Mateusz Kozłowski, Katarzyna Nowak, Aneta Cymbaluk-Płoska

**Affiliations:** Department of Gynecological Surgery and Gynecological Oncology of Adults and Adolescents, Pomeranian Medical University, 70-111 Szczecin, Poland; kn13222@gmail.com (K.N.); aneta.cymbaluk@gmail.com (A.C.-P.)

**Keywords:** ovarian cancer, BRCA1 mutation, PARP inhibitor, olaparib

## Abstract

Ovarian cancer is one of the most common cancers of the reproductive organs. As there are no symptoms in the early stages, it is mainly detected in the advanced stages. Even then, the symptoms are non-specific and include, for example, abdominal pain, early satiety, or changes in bowel habits. Both biochemical marker levels and imaging studies are used in the initial diagnosis. However, it should be emphasized that they are not characterized by high specificity. Treatment is multistage, and usually first-line debulking surgery is used followed by platinum-based chemotherapy. Here we present a clinical case of a 56-year-old female, a carrier of a mutation in the BRCA1 gene, with a history of breast cancer and with recurrent epithelial ovarian cancer. The patient was qualified for treatment with a PARP inhibitor and is currently undergoing treatment with olaparib. In the patient’s follow up of 50 months to date, there has been no recurrence of cancer. Few side effects have been observed, and the most serious one that can be effectively treated is anemia. On the basis of the described case, the authors concluded that olaparib treatment is effective, relatively safe, and does not significantly affect daily functioning.

## 1. Introduction

Globally, ovarian cancer is the eighth most common cancer among women according to WHO data. It is ranked eighth in Europe and eleventh in the United States. It is the eight most common cause of cancer death [1]. Ovarian cancer is one of the most challenging cancers to diagnose early. This is due to the lack of screening tests and the presence of non-specific symptoms or complete absence of symptoms in the early stages of the disease [2]. Therefore, at the time of diagnosis a tumor is ordinarily advanced. Only a quarter of patients are diagnosed at stage I or II, almost 60% are diagnosed at stage III, and 17% at stage IV [3]. Blood tests, tumor markers such as cancer antigen 125 (CA 125) or human epididymis protein 4 (HE4), and transvaginal ultrasound are used for diagnosis. There are also several algorithms such as the RMI (Risk of Malignancy Index) or the ROMA (Risk of Ovarian Malignancy Algorithm) used in the diagnostics of ovarian cancer [4,5]. Certain diagnosis is mainly based on the result of histopathological analysis of the material collected from the tumor during primary surgery. Treatment options for ovarian cancer include surgery, chemotherapy, radiotherapy, targeted therapy, and immunotherapy [6]. Debulking surgery with platinum-based chemotherapy is usually used as a first-line treatment [7]. A relatively new, innovative method used in the treatment of ovarian cancer is the usage of PARP inhibitors (Poly(ADP-ribose) polymerase (PARP) inhibitors) [8]. Their mechanism of action is to disrupt DNA repair at several key points [9]. One of the PARP inhibitors which is approved as a therapy for ovarian cancer is olaparib. It is used for the maintenance treatment of recurrent ovarian cancer in patients carrying mutations in the BRCA1 or BRCA2 genes who have responded completely or partially to platinum-based chemotherapy [10].

Here we present a clinical case of a 56-year-old female, a carrier of a mutation in the BRCA1 gene, with a history of breast cancer, who is undergoing chemotherapy with a PARP inhibitor—olaparib. The patient is treated in the Department of Gynecological Surgery and Gynecological Oncology of Adults and Adolescents, Pomeranian Medical University in Szczecin (hereinafter referred to as the Department).

## 2. Case Report

In a 56-year-old Caucasian female patient, menarche occurred at the age of 14, and last menstruation at the age of 44, which was associated with hysterectomy. The patient had two single pregnancies, twice delivered by caesarean section. The patient’s mother had breast cancer, and the patient’s grandmother (from her mother’s side) had ovarian cancer. 

At the age of 35 the patient was diagnosed with right breast cancer and underwent right-sided mastectomy with postoperative chemotherapy and radiotherapy. Due to the presence of a germline mutation in BRCA1 gene, the patient underwent prophylactic left mastectomy with left breast reconstruction at the age of 37 and right breast reconstruction one year later. At the age of 44, during a follow-up gynecological ultrasound a tumor of the right adnexa region (7 cm) was found; in addition, the CA125 level was elevated (44 U/mL). At that time, the patient felt more tired than usual. It should be noted that in an ultrasound performed 10 months prior, this tumor was not visible. A laparotomy was performed during which the uterus, bilateral adnexa, and the omentum were removed. On histopathological examination, carcinoma endometrioides G2 was found in the left ovary; the left fallopian tube showed no neoplastic changes, and carcinoma endometrioides G2 was found in the right ovary and a tumor collected from the area of right adnexa (7 cm). The right fallopian tube was free of neoplastic changes; no neoplasmatic infiltration were found in the uterus or in the omentum. After surgery, the CA125 level was within normal limits. Oncological treatment also included chemotherapy based on cisplatin and paclitaxel (six cycles). Remission of the disease was achieved. Six years later, the ovarian cancer recurred. This time the patient was again experiencing increased fatigue. In control blood tests, an increased level of tumor markers weas observed: HE4 = 280.1 pmol/L, CA125 = 113.3 U/mL, ROMA = 77.4%. A small amount of fluid in the peritoneal cavity was present during an ultrasound examination. Abdominal CT (computed tomography) scan showed multiple implants in the parietal peritoneum, which was confirmed by a PET (positron emission tomography) scan. Because of the inoperable nature of the lesions, six cycles of chemotherapy based on cisplatin and paclitaxel were introduced. A tumor marker panel was performed prior to the administration of each cycle. Finally, a significant reduction in tumor markers levels was achieved: HE4 = 79.9 pmol/L, CA125 = 15 U/mL, ROMA = 17.5%. Remission of the disease was achieved. One year after completing chemotherapy, the patient again reported fatigue. A follow-up CT scan of the abdomen and pelvis showed no tumor recurrence. PET was also performed and showed the presence of two tumors of the sigmoid region. Based on this examination, re-recurrence of the cancer in the recto-vaginal space was suspected. Consequently, the patient underwent colonoscopy with collection of colonic mucosal specimens. No macroscopic changes were observed during the examination; the histopathological examination of the specimens did not reveal any neoplastic changes. The patient was qualified for surgery with subsequent chemotherapy. A sigmoid resection with reconstruction of the gastrointestinal tract continuity was performed. Histopathological examination yielded a diagnosis of serous carcinoma. In addition, tumor metastases were found in seven resected lymph nodes. The patient then underwent HIPEC (hyperthermic intraperitoneal chemotherapy). The next stage of treatment was implementation of chemotherapy—six cycles of cisplatin and paclitaxel. Tumor marker tests performed before each cycle showed a decreasing trend. A CT scan of the abdomen and pelvis showed no metastatic foci or recurrence. 

**Figure 1:**
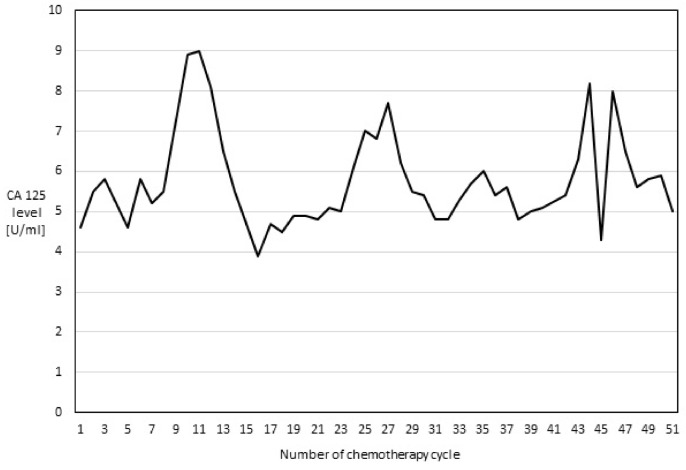
CA125 level in relation to olaparib chemotherapy cycle.

In January 2017, the patient was qualified for maintenance treatment with the PARP inhibitor—olaparib, and thus received the first cycle of the relatively novel, oral chemotherapy. The administration of the drug takes place during hospitalization, on average every 28 days. The daily dose of olaparib is 800 milligrams. It is worth noting that the time of a standard hospitalization with qualification and dispensing of the drug is one day. At each hospitalization, a set of laboratory blood tests is performed. Based on the results, the patient is qualified for drug administration. The panel includes blood count, alanine aminotransferase, aspartate aminotransferase, total bilirubin, CA125, and creatinine. CA125 measurements allow the monitoring of treatment for possible recurrence. In the patient, no significant increase in the level of cancer marker 125 was observed throughout the treatment, as shown in Figure 1. 

**Figure 2:**
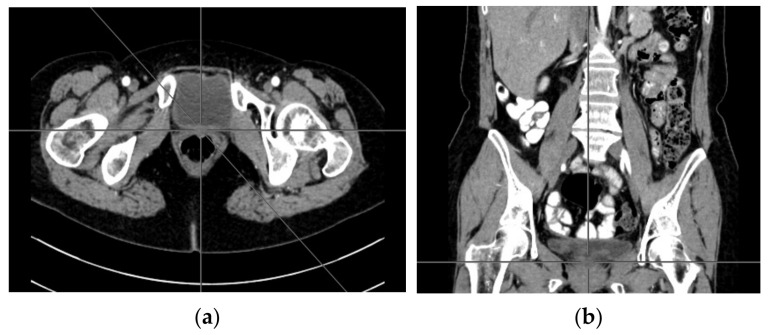

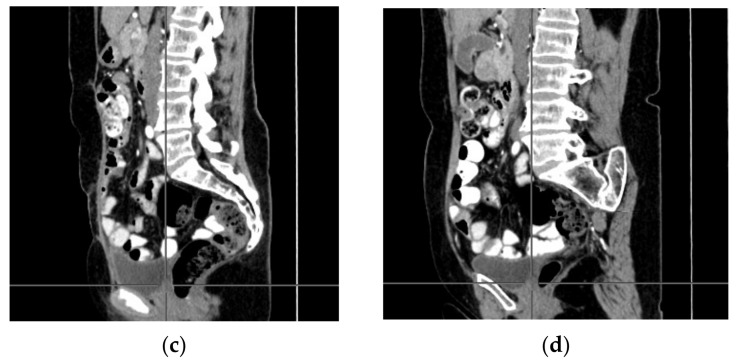
(**a**–**d**) CT scans performed at the beginning of olaparib treatment.

**Figure 3:**
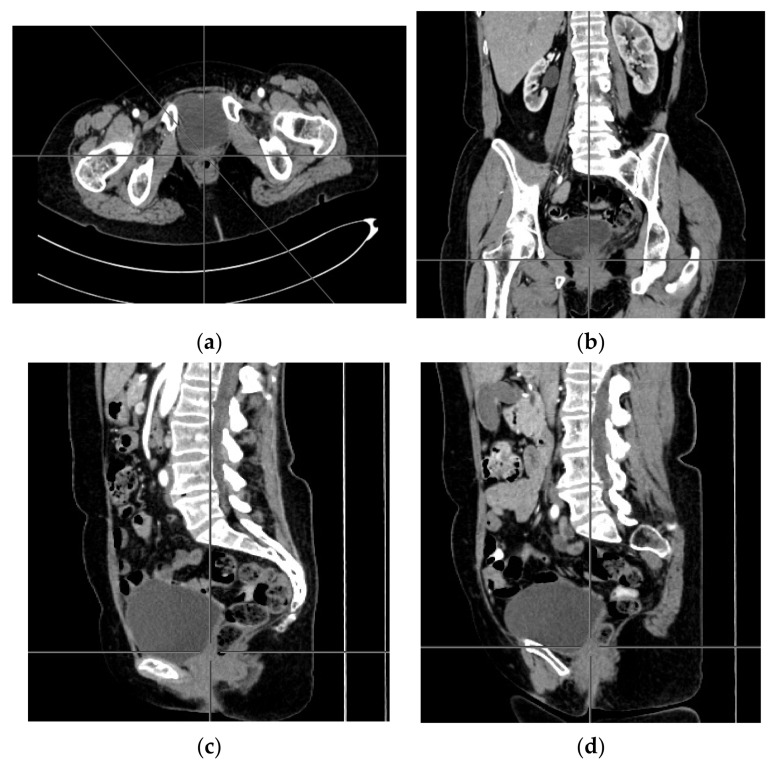
(**a**–**d**) CT scans performed before 48 cycle of olaparib treatment.

A computer tomography of the abdomen and pelvis was also performed every 3 months for the possible detection of ovarian cancer recurrence. No CT changes suggestive of recurrence were observed in the patient during the treatment. There are illustrates scans on the same section from CT performed at the beginning of PARP inhibitor treatment (Figure 2) and before 48 cycle of olaparib chemotherapy (Figure 3). 

The monitoring of marker levels and imaging studies showed a long period of time without relapse. The patient had anemia during treatment (Haemoglobin (Hgb), approximately 6.5 mmol/L; red blood cell count (RBC), 2.5 T/L; Haematocrit (Hct), 0.3 l/L). For this reason, the patient is currently treated with oral iron preparations. Anemia was also the cause of several disqualifications from chemotherapy administration. Apart from that, the patient experiences mainly mild side effects, such as fatigue, periodic nausea, occasional joint pain, and back pain. Since the beginning of treatment with PARP inhibitors, the patient has been under the constant care of the Department. To this day, the patient has received 51 cycles of chemotherapy, and the duration of treatment is 50 months (4 years and 2 months).

## 3. Discussion

Ovarian cancer, due to its poor symptomatology, is mostly detected at an advanced stage [11]. Typically, symptoms are nonspecific and include abdominal bloating, abdominal pain, urinary frequency, early satiety or feeling full, or changes in bowel habits, which can delay diagnosis [2]. It was fatigue that was the first symptom noticed by the described patient. It should also be noted that current diagnostic tools are not sensitive enough to detect ovarian cancer at an early stage. The primary diagnosis is based on blood levels of HE4 and CA125 and transvaginal ultrasound. Based on these parameters, tools have been developed that are currently used in diagnostics [5]. The first is RMI, which was originally proposed in 1990 by Jacobs et al. [12]. In addition to CA125 and ultrasound findings, it also considers menopausal status. The second tool is ROMA, which is associated with HE4 and CA125 levels according to the menopausal status [13]. It was the image of the tumor on ultrasound and the elevated CA125 level, along with the accompanying nonspecific symptoms, that provided the indication for surgical treatment. Due to the presence of a BRCA1 gene mutation, history of breast cancer, and mastectomy, the patient received annual gynecological follow-ups. Due to that, the tumor was spotted on ultrasound, despite the sparse symptoms. The final diagnosis was made on the basis of histopathological examination of the resected tissue. The most common ovarian malignancy is epithelial ovarian cancer (EOC) [14], and the most common histologic type is high-grade serous ovarian cancer [15]. Endometrioid carcinoma, which was diagnosed in the patient, accounts for approximately 10% of EOCs. It is interesting to note that in patients with the BRCA1 mutation, the most common ovarian cancer is HGSOC [16]. Surgical treatment of patients depends on the clinical stage which is documented using the International Federation of Gynecology and Obstetrics (FIGO) and TNM staging classifications [17]. In most cases, the main goal of treatment is to perform cytoreductive surgery. The next stage of treatment is first-line chemotherapy consisting of a platinum derivative and paclitaxel. This is exactly the treatment that was given to the patient. After primary oncologic treatment, patient care consists of regular measurement of ovarian cancer markers and CT scans of the abdomen and pelvis. The classification of patients for second-line treatment is based on sensitivity to platinum derivatives used in first-line therapy. Patients are divided according to the tumor’s response to first-line treatment and PFS (progression-free survival): 1. platinum-refractory—progression during first-line treatment, 2. platinum-resistant—recurrence within 6 months from completing first-line treatment, 3. partially platinum-sensitive—recurrence within 6–12 months from completing first-line treatment, 4. platinum-sensitive—recurrence more than 12 months from completing first-line treatment [18,19]. In the described patient, the recurrence of the cancer occurred six years after the completion of treatment. According to the above classification, the patient belongs to the “platinum-sensitive” group. It should be noted that despite such spread of neoplastic foci, the patient reported only fatigue as a symptom. A 6-cycle chemotherapy based on cisplatin and paclitaxel was started again. After this treatment, the remission time was shorter—1 year. Interestingly, CT images showed no lesions, while PET showed two tumors of the sigmoid region. Resection of the tumors along with the sigmoid colon showed the presence of serous carcinoma on histological examination. Interestingly, the histological picture was different from that of the primary ovarian tumor. The patient was also treated with HIPEC followed by 6-cycle chemotherapy with cisplatin and paclitaxel. Randomized clinical trials have investigated the feasibility and benefit from adding a cycle of HIPEC during surgery [20,21]. It was demonstrated that HIPEC was feasible and tolerable [21]. The median recurrence-free survival was shorter in the surgery group compared to the surgery-plus-HIPEC group (10.7 months vs. 14.2 months). The median OS (overall survival) was 33.9 months in the surgery group and 45.7 months in the surgery-plus-HIPEC group [21].

**Figure 4:**
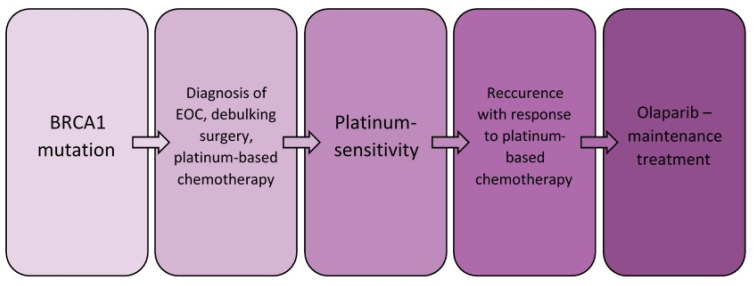
Important points in the diagnostic and therapeutic management of the described patient.

The population risk of ovarian cancer is 1. 3% to 1. 9% [22], while by age 70 for BRCA1 mutations it was about 40%, and for BRCA2 mutations it was 18% [23]. Both BRCA1 and BRCA2 proteins play a key role in preventing the cancer process. They participate in the process of DNA double strand break (DSB) repair by regulation of homologous recombination (HR) [24]. The cellular effects of BRCA1/2 mutations are the target of relatively recently introduced new anticancer drugs—PARP inhibitors. In general, the multidirectional effects of PARP inhibitors are inhibition of BER (Base Excision Repair), trapping of PARP-DNA complex, enhancement of NHEJ (Non-Homologous End Joining), and the inhibition of Alt-EJ (Alternative End Joining) [25]. BRCA1/BRCA2 mutations include germline and somatic mutations. An analysis of Study 19 shows that the benefits of olaparib treatment for patients with somatic mutations are similar to those obtained in patients with germline mutations [26]. Similar results of ovarian cancer treatment in patients with different types of BRCA1/BRCA2 mutations treated with one of the PARP inhibitors, rucaparib, have been described by Swisher et al. [27]. Based on the SOLO2 trial, which used olaparib in the treatment in BRCA-mutated relapsed ovarian cancer patients after complete or partial response to platinum chemotherapy, the therapy was found to be highly effective [28]. In 2018, olaparib was approved by the Food and Drug Administration (FDA) for first-line maintenance treatment in ovarian cancer, in patients who have a mutation in the BRCA1 or BRCA2 gene, with partial or complete platinum sensitivity [29]. Analyzing both clinical trials raises the question of when to include olaparib in ovarian cancer therapy. Whether to wait for a recurrence to occur, or to implement platinum-based chemotherapy as a continuation. As the SOLO1 study is still ongoing, an answer to this question is still to be awaited. According to Recommendations of the Polish Gynecological Oncology Society for the diagnosis and treatment of ovarian cancer, In Poland there was a program of olaparib therapy, according to which it was required to start the therapy within 8 weeks from the last administration of platinum-based chemotherapy [19]. The patient was qualified for treatment with olaparib. The advantages of this chemotherapy are certainly the oral route of administration and shorter hospitalization time, which had a positive effect on the patient’s well-being and did not increase the feeling of illness. Attention should be paid to the effectiveness of treatment in the patient. Throughout her treatment with olaparib to date, the patient has had oncologic follow-up in the form of CA125 level determination and a follow-up CT scan. There was no increase in CA125 for more than 50 months of treatment; additionally, the CT scan did not show changes suggestive of recurrence. This confirms the results of clinical trials, according to which introduction of olaparib to the first-line treatment allows one to achieve a significant progression-free survival improvement with no detrimental effect on quality of life [28,30]. During the treatment, however, the occurrence of adverse events is observed, which can be divided into two groups: haematological and non-haematological. The most common of the first group include anemia and neutropenia, while the second group include nausea and fatigue or asthenia [28]. It should be noted that in the patient, despite a long period of treatment, adverse events are observed, but they are relatively few. The following symptoms, mentioned in the SOLO2 study as the most frequent, were mainly observed: anemia and fatigue; nausea, arthralgia, and back pain occurred sporadically. The current course of the patient’s disease indicates that ovarian cancer is a chronic disease with periods of remissions and recurrences. The described case demonstrates personalized and individualized management of the patient. Implementation of specific treatment with PARP inhibitor deserves attention—this treatment can be performed only in a specific group of patients. The presence of the BRCA1 mutation, platinum sensitivity, and the recurrence of cancer after platinum derivative therapy were the criteria for olaparib chemotherapy. In the diagnostic and therapeutic process, these key stages should be indicated, as shown in Figure 4. 

Long duration of treatment with olaparib with very good response, no recurrence and good quality of life, including psychological state, are the essence of oncological treatment.

## 4. Conclusions

Olaparib treatment is effective; the patient has not had a tumor recurrence for 4 years and 2 months. This gives hope for a long survival despite such a serious disease. It is also relatively safe—side effects are minor. As for one of the more serious side effects, anemia, we can treat it effectively. Due to the relatively infrequent and short duration of hospitalization, treatment with olaparib does not considerably affect daily functioning. It affects significantly less the performance of daily duties and social relationships and does not increase the sense of illness. The route of administration of the drug is beneficial and not very traumatic for the patient.

## Data Availability

Not applicable.
